# Validity and Reliability of an Assessment Tool for the Screening of Neurotoxic Effects in Agricultural Workers in Chile

**DOI:** 10.1155/2019/7901760

**Published:** 2019-10-30

**Authors:** Boris Lucero, Paula A. Ceballos, María Teresa Muñoz-Quezada, Carolina Reynaldos, Chiara Saracini, Brittney Olivia Baumert

**Affiliations:** ^1^The Neuropsychology and Cognitive Neurosciences Research Center (CINPSI Neurocog), Faculty of Health Sciences, Universidad Católica del Maule, Talca 3460000, Chile; ^2^Department of Nursery, Faculty of Health Sciences, Universidad Católica del Maule, Talca 3460000, Chile; ^3^Department of Environmental Health, Rollins School of Public Health, Emory University, Atlanta, GA 30322, USA

## Abstract

There is a substantial use of pesticides within the agricultural industry of Chile, with neurotoxic effects through mechanisms of acetylcholinesterase inhibition. These pesticides result in deterioration in health, increasing the risk of diseases such as Parkinson's and Alzheimer's in highly exposed occupational population. To date, there are no brief assessment tools to monitor cognitive impairment in agricultural workers chronically exposed to these pesticides. *Method*. 234 agricultural workers and 305 nonagricultural workers were assessed two times (test-retest) through a brief tool which comprised three tests (clock-drawing test (CDT); frontal assessment battery (FAB); trail making tests (TMT) A and B). The full scale of WAIS-IV was administered as a gold standard to 18% of the sample of agricultural workers. Factor analysis was used to evaluate the factor structure, and validity and test-retest reliability were assessed concurrently. *Results*. Cronbach's alpha values were satisfactory or above (>0.60). Test-retest correlations were all significantly correlated (*p* < 0.001). All the tests had a significant correlation with the full scale IQ score of WAIS-IV (*p* < 0.05). The Kaiser–Meyer–Olkin (KMO) measure was 0.74, and the Bartell sphericity test = *p* < 0.001. Three factors explaining 61.62% of the variance were extracted. Two items of the FAB test were dropped of the final factor solution. Normative data transformed into percentile scores and stratified by age and educational level were obtained for Chilean agricultural workers. *Conclusion*. The brief assessment tool has adequate metric properties as a screening instrument. This allows for a simple administration test (10 to 15 minutes) that can potentially be used for the rapid monitoring of cognitive deterioration in the face of occupational exposure to pesticides in agricultural workers.

## 1. Introduction

Adverse health outcomes from pesticide-related exposure are a global issue impacting both industrialized and developing countries [1, 2]. There is evidence that those most critically impacted by the adverse health implications from pesticide exposure are those in the occupational agricultural setting [3–8].

In Chile, the most frequently sold group of pesticides is the organophosphates (OP), specifically diazinon and chlorpyrifos [9]. The primary mode of action of OP pesticides is via the enzyme acetylcholinesterase [10]. The inhibition of acetylcholinesterase generates an excessive excitation of the muscarinic and nicotinic receptors of the nervous system, causing the acetylcholine neurotransmitter to overaccumulate in the cholinergic synapses. This eventually results in acute or chronic intoxication. Studies found that the neurotoxic effect of pesticides can provoke decreased cognitive performance in developing children and can be considered a precursor to an increased risk of degenerative diseases in chronically exposed workers in occupational settings [3, 11, 12]. In the last decades, increased attention has been paid internationally to the development and administration of tools which can evaluate the neurobehavioral and cognitive effects of the exposure to neurotoxic substances such as pesticides [5–7]. The administration of these kinds of tools to populations of agricultural workers exposed to chemicals has been developed for decades using methods derived from neuropsychology and experimental psychology. Usually, batteries of a series of behavioral tests have been used to evaluate the effects of neurotoxic pesticides, and the number of tests to be used in these batteries has increased over time [7, 10, 13]. One of the most important tools is the Neurobehavioral Core Test Battery (NCTB) [14]. It is composed of seven behavioral tools: Digit Symbol test, Digit Span test, Simple Reaction Time, Benton Visual Retention test, Santa Ana Dexterity test, Pursuit Aiming II, and the Mood Profile [15, 16]. Noteworthy, the selection of the tests composing the NCTB battery has been performed on the basic rule that these must be proven to be tools sensitive to the changes associated with exposure to chemicals. It means that the tests have shown to be effective in differentiating between groups of workers who had been exposed or not to chemicals, allowing the identification of neurotoxin effects. This implies that batteries also had to comply with the two basic psychometric properties of validity and reliability. In this regard, in 1999 [17], one of the main recommendations for NCTB was the need to establish construct validity in a wide range of different countries. In fact, it had been previously concluded that this battery could effectively assess only the adult population with twelve or more years of education and belonging to North America, Western Europe, and some parts of Asia, while it did not show the same reliability of use on people with less than nine years of education, being additionally discarded its effective use in populations different from the North American and European. In 2013, different aspects of the use of the battery and the selection criteria of the tests for the measurement of neurotoxic effects were discussed again [18]. For example, it was recommended that the elaboration of such type of battery should not include copyrighted tests. This was a relevant point, since most of the tests included in the NCTB were copyrighted; in fact, they should be acquired individually in order to be used legitimately. Instead of this, experts recommend including tests that are of public domain, such as the trail making test (TMT) [19]. A final conclusion regarding the tests is that they should be free of cultural influence. Experts furthermore agreed that it is always necessary to have a control group for comparison in epidemiological studies of neurotoxic exposures. Particularly in the case of the workers, it was pointed out that it was necessary to apply tests in a test and retest context, in order to establish an early monitoring of the occupationally exposed populations with respect to the neurotoxic effect on a follow-up logic. Finally, they concluded that it is necessary to develop a dedicated “screening” assessment tool made up of sensitive tests to be used in the field of human neurotoxicology [18]. Other authors pointed out that the neuropsychological tools that result in a greater effect size with respect to OP pesticides are those that measure working memory/attention, visual memory, psychomotor speed, executive function, and visuospatial ability [5]. Therefore, it can be concluded that the cognitive functions affected to a greater extent by the exposure to OP pesticides are nonverbal abilities, being found in cross-sectional studies a slowdown of reaction time and deficits in the performance in short-term memory and executive function in the most severely affected individuals [5]. Moreover, a constant monitoring of the agricultural workers' neurocognitive functioning is necessary, due to the evidence that pesticides increase the risk for neurodegenerative diseases, such as Parkinson's [20] or dementia and Alzheimer's disease (AD), as it has been observed in longitudinal cohort studies [21].

Different types of neurotoxic disorders have been associated with chronic OP exposure: (1) cholinergic syndrome; (2) intermediate syndrome; (3) OP-induced polyneuropathy; (4) chronical exposure-related neuropsychiatric disorders. The first two disorders are generally detected in case of acute intoxication in the emergency care hospitals and are immediately treated and registered. However, the third and fourth kind of diseases need a detailed monitoring, for example, through a screening assessment tool, and their symptoms may appear later in the individual's life, with a progressive deterioration of the neurobehavioral performance, associated to permanent damages in the central nervous system (CNS), and cognitive deficits, including memory, concentration and learning, attention, information processing, and reaction times.

In Chile, there are few studies in the area collecting evidence of neurocognitive risk in the agricultural workers, the most complete and relevant being the one carried out in the Maule region [22]. In this study, full intelligence scales (Wechsler Adult Intelligence Scale, WAIS-IV) [23] were used to measure neurocognitive effects. The use of this kind of tools, although is advisable in terms of a more specific notion of the performance and the cognitive profile of test takers, implies a longer time of both test administration and further interpretation of the results. These “practical” features make it difficult to adopt them as screening and monitoring tests considering the amount of human and financial resources needed to perform the study, both from an organizational and individual point of view. Therefore, in the Chilean context, nationally validated tools (such as batteries of tests) able to show neurological problems in agricultural occupational populations which are at the same time sensitive and viable for a fast and easy administration are still missing. For this reason, it appears clear that it is necessary to establish an economic test, easy to apply and validated at a national level with agricultural occupational population for the early monitoring of the cognitive effects of exposure to acetylcholinesterase inhibitor pesticides. This test should be directly and specifically focused on the evaluation of these cognitive areas that have been recognized as more sensitive in the adverse effects on workers' health (reaction time, short-term memory, and executive functions), in order to perform an effective surveillance in the context of occupational exposure. Accordingly, the aim of this study is to validate a brief assessment tool for the monitoring of neurotoxic effects in agricultural workers exposed to pesticides, which can be used as a screening measure for the early detection of cognitive impairment in the occupational context.

## 2. Materials and Methods

### 2.1. Participants

**Figure 1:**
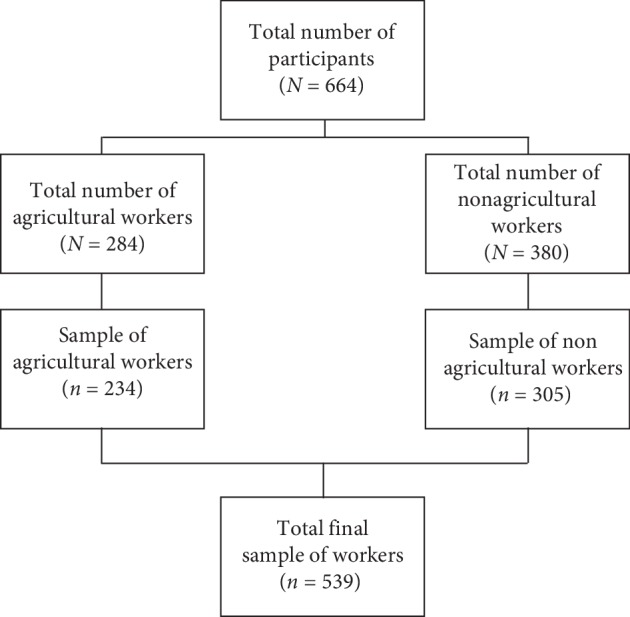
Study participants and sample of agricultural and nonagricultural workers.

A sample of 664 workers participated in this study between 2017 and 2018. Of these, 284 were agricultural workers from the Maule region, and 380 were nonagricultural workers from the Metropolitan and Valparaiso regions (see Figure 1). For the reliability analysis, a subsample of 193 agricultural workers and 193 nonagricultural workers (total *N* = 386) had been administered both the test and retest (72% of the total sample). Each participant has signed an informed consent document prior to the beginning of the study. The research protocol and the informed consent documents have been revised and approved by the certified Ethics Committee of the Safety Mutual CChC.

Workers of the exposed group (*n* = 284) were recruited from agricultural companies of the Maule region that accepted to participate in the study, and they were randomly chosen from those who met the inclusion criteria. Inclusion criteria were that participants must be permanent agricultural workers, older than 18 years old, with at least 2 years of occupational exposure to pesticides, and should have normal healthy condition at the moment of the study. Exclusion criteria, on the other hand, were that workers were temporary, younger than 18 years old, and had any health condition (metabolic diseases, or any which could alter the interpretation of results). All this information has been collected through an initial “Pesticides Exposure Questionnaire” already validated in Chile from a previous study [22].

Workers of the control group (*n* = 380) were randomly selected from building companies of other regions (Santiago and Viña del Mar) far from agricultural fields and crops. Inclusion criteria were that they should not be agricultural workers, older than 18, that had not worked (neither temporally) in agriculture for the last 5 years, and that have not been occupationally exposed to any neurotoxic. This information has also been collected by a previous questionnaire.

After a first cleaning process, filtering all the missing data and eliminating subjects that did not meet the inclusion criteria, the sample reached a number of *N* = 539 for the analysis (see Figure 1).

### 2.2. Materials

#### 2.2.1. Tests Selection

With the aim to select the most appropriate tests for the brief assessment tool, the content validity has been assessed through an accurate literature review and construct identification. We found different tests of processing speed, attention, and visual memory. In a second phase, an evaluation of the tools by three expert referees' with an average work experience in the field of neuropsychological evaluation of 14 years has been carried out. Based on the results of these two phases, the clock-drawing test (CDT), the frontal assessment battery (FAB), and the trail making tests (TMT) A and B have been chosen to be included in the proposed brief assessment tool. Each of these tests is described in the following sections, and a summary of the functions evaluated by them is presented in Table 1.

#### 2.2.2. Clock-Drawing Test (CDT)

The clock-drawing test is a simple paper-and-pencil task which takes a short time to be administered, corrected, and scored. Basic instruction given to the participant is to draw an analog clock displaying the 11 : 10. For the scoring, 20 items are considered, giving 0 or 1 points depending on the accomplishment of what is indicated for each item. The maximum total score is 20. Based on the study from Mendez and colleagues [24], the binary items could be grouped in aggregated scores. The authors established three groups of items by correlating the scores with other neuropsychological tests. The first group has been strongly associated with visuoperceptual measures. The second group has been associated with attention measures. The third group has not been associated with any correlated test in the aforementioned study and has been considered with items measuring numerical sequencing. In our study, we attained to this grouping, producing three subscores: visuospatial skills (CDT-VS), attention (CDT-ATT), and numerical sequencing (CDT-NS), plus an aggregated total score based on the 20 total score. Three or more errors have been considered as cognitive impairment, and normal subjects show 2 errors or below.

#### 2.2.3. Frontal Assessment Battery (FAB)

The frontal assessment battery is a brief test taking no more than 10 minutes to be administered. It is composed of 6 items exploring different abilities (see Table 1) associated to the frontal lobe functions, allowing the identification of executive dysfunctions, and the early detection of diseases such as Alzheimer's, Parkinson's and progressive supranuclear paralysis (PSP). Each item can receive a score from 0 to 3 points. Authors only give a general interpretation of the tool, which is relative to the global score of FAB test, allowing evaluating the severity of the executive dysfunction and obtaining a descriptive pattern of the worse area according to the specific items showing a worse performance.

#### 2.2.4. Trail Making Test (TMT)

The trail making test is composed of parts A and B. TMT-A requires the subjects to draw continuous lines connecting 25 scrambled numbered circles following the numeric sequential order (from 1 to 25). Similarly, TMT-B requires the participant to draw a continuous line connecting alternatively the circles with numbers and those with letters following each its own sequence (i.e., 1, A, 2, B, 3, C, etc.). In both versions, the examiner instructs the subjects to never lift the pen from the paper, and if he/she makes an error, he/she has to return to the circle where the error originated and continue. The final score is represented by the total time (seconds) needed to complete the task. Accordingly, a lower score in TMT expresses a better performance, while longer times mean that the subject had problems completing the task. As a cutoff for abnormal performance, commonly 78 or more seconds for TMT-A and 273 or more seconds for TMT-B for completion time are considered.

Besides the tools described above, the Chilean-standardized Spanish version of the Wechsler Adults Intelligence Scale (WAIS-IV [23]) has been administered to the 18% of the agricultural workers sample (*n* = 41) as a gold standard to compare performances obtained with the brief assessment tool. The administration of this test took between 1 hour and half and 2 hours for each worker.

### 2.3. Procedures

Assistants received an intensive training in the Laboratory of the Neuropsychology and Cognitive Neurosciences Research Center (CINPSI Neurocog) in the Catholic University of Maule (UCM, Talca, Chile) previously to the beginning of all the test administrations.

Before starting the administration of the brief assessment tool, a preliminary pilot assessment including the whole set of tests has been given to 6 agricultural workers in order to fix any problems with the tool's performance. From the analysis of the performances of this preliminary sample, it was verified that the tests worked fine and did not need any change or modification to the compiled administration protocol.

The final assessment protocol has been administered to test takers in two times, with an intermediate lapse of 2–4 weeks. All tests have been given in their workplaces, and each company facilitated an appropriate space (without too much noise or interferences) for test administration. Each assessment was performed by an assistant to a worker at a time. The test taker had to read the informed consent and eventually sign a participation agreement. Then, the assistant explained briefly the sequence of actions he/she was going to perform and answered his/her eventual questions.

### 2.4. Analyses

Statistical analyses have been performed with R-Studio [26] and SPSS 24.0 [27]. Before starting the analyses, a coresearcher and a research assistant checked 100% of the protocols for recording integrity and correctness of scoring. Firstly, an extensive exploratory analysis has been performed to individuate all the missing data in the examined variables. After that, the variables have been checked for normal distribution with the Shapiro–Wilk test. On the basis of the results of this analysis (all the tests had a *p* value less than 0.001), nonparametric statistical tests were performed for the following analysis of data. Nonparametric correlations (Spearman's rho) have been performed on the test-retest evaluation, while Cronbach's alpha coefficient has been calculated to measure internal consistency of the brief assessment tool. For the construct validity test, a factor analysis has been performed, by means of which the eigenvalues >1 have been extracted with a maximum of 25 iterations to obtain convergence. Also, a direct oblimin rotation method has been applied to generate the rotated factor solution, considering the same 25 iterations to converge. The Mann–Whitney *U* test has been performed for the means comparisons between the two groups. An analysis of the convergent validity was made with a subsample of 41 workers to whom the WAIS-IV test was administered as a Gold Standard measure. This test is an intelligence scale composed of 10 subscales, which yields a total score that after being transformed to the standard score represents the intellectual quotient (IQ) of the test takers. In addition to the above, the estimation of the normative values of the brief scale by age group and educational level was made from the percentiles represented by each stratified score.

## 3. Results and Discussion

### 3.1. Descriptive Analysis

For this analysis, 539 workers have been considered (agricultural, *n* = 234; nonagricultural, *n* = 305). From the agricultural workers sample, 28% of them currently apply pesticides and 37.2% made the last application during 2 years or less. A 28% of this sample applies pesticides only as a temporary job, while 13.7% does it permanently. A 27% applied pesticides since less than 10 years, while 21.4% applied since 10 years or above. A 33.3% declares to have a certification of pesticide applicator. A 57.7% declare to be aware of the health hazards of the application of pesticides, and 50.4% stated that they have received training on these hazards. A 42.3% declare wearing personal protective equipment (PPE) during their work when they mixed the pesticides, and a 44% said that they changed their clothes after apply chemicals at work. Only 7.7% referred that they have been intoxicated and 3.4% that they were hospitalized after pesticides poisoning. With regard to symptoms, a 15% have experienced dizziness, nausea, fatigue, vomiting, and/or salivation while applying pesticides. A 38.5% had a cholinesterase test, and only 3.8% had an exam that indicated severe poisoning. Demographic characteristics of each group composition are shown in Table 2.

Also, descriptive statistics about the scores obtained for each group in each administered test have been calculated (see Table 3).

### 3.2. Content Validity

As described above, construct domains have been qualitatively identified during the tool development phase through an extensive literature review that allowed to find tests that were brief, were of public domain, and addressed the assessment of the cognitive functions of importance related to the neurotoxic effects of the most used pesticides in Chile. Then, they were revised by a pool of experts on the field of neuropsychology to evaluate their content validity. The expert judges totally agreed that CDT measured visuospatial skills (100% agreement), while partially agreed that this test measures focused attention, planning and organizational abilities, and inhibitory control. With respect to the FAB, they completely agreed (100%) that it measures planning and inhibitory control and partially agreed (75%) that it measures working memory, sustained attention, and organization ability. Regarding TMT-A, there was total agreement (100%) that it measures processing speed and partial agreement (75%) about focused and selective attention, planning, inhibitory control, and visuospatial skills. Finally, in the case of TMT-B, judges agreed (100%) that it consists in a working memory and sustained attention measurement and partially agreed (75%) that it is a self-monitoring measurement. From these interjudge evaluations, the test of the brief assessment tool with strongest relative weight was the FAB, and progressively lower weights resulted for TMT-A, CDT, and TMT-B. The cognitive function of simultaneous processing did not reach agreement for any evaluated test and therefore is not considered as a construct included in the brief assessment tool, while focused attention and self-monitoring reached the lowest interjudge agreement level. On the other hand, the highest agreement values have been reached by visuospatial skills, selective attention, planning, processing speed, and inhibitory control.

### 3.3. Internal Consistency

Internal consistency analyses have been performed calculating Cronbach's alpha for each subtest, except TMT-A and TMT-B, since their scores represented times of completion and were not comparable. CDT has an alpha = 0.838, indicating a high internal consistency for its 20 items. FAB's initial analysis on all the 6 items had an alpha = 0.606; eliminating the item 6, internal consistency reached alpha = 0.633, considered as satisfactory value. For this reason, in the following analyses, this item has been not considered.

### 3.4. Construct Validity

**Figure 2:**
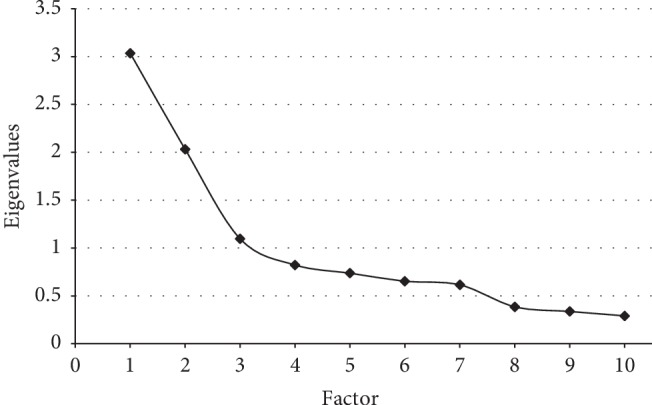
Screen plot with the eigenvalues of each factor yielded from the factor analysis.

Construct validity has been calculated with factor analyses on scores of the entire sample (*N* = 539). A number of 10 variables have been considered as input variables, including the 3 CDT aggregated items (visuospatial, attention, and numerical sequencing), 5 FAB items, and TMT-A and TMT-B scores. The sample obtained a KMO = 0.742 at the Kaiser–Meyer–Olkin value (recommended value is 0.6). The Bartlett's sphericity test was applied and resulted in a value of *χ*^2^ (45) = 1291, *p* < 0.001, which indicates that the correlation structure is adequate to perform factor analyzes. The principal axis factoring (PAF) was used as the extraction method, since the data of the tools were not normally distributed [28]. Then, the instrument was subjected to a direct oblimin rotation method with a cutoff of 0.30 and using the Kaiser's criterion of eigenvalues greater than 1, which yielded a three-factor solution as the best fit for the data, accounting for 61.62% of the variance. The factors four to ten resulted with eigenvalues below 1 (Figure 2), only explaining between 8% and 2% of variance. The results of this factor analysis are summarized in Table 4. The item 2 of the FAB was eliminated, since it was not integrated into any of the factor solutions tested, with factor weight less than 0.30.

The representation of the resulting three-factor solution was made using the labels that emerged from the evaluation of expert judges with respect to the skills measured by the tests included in the brief assessment tool. After the interpretation of the components as described, the following descriptors were obtained for each one: (a) visuospatial skills and processing speed: this factor had an eigenvalue of 3.03 and accounted for 30.34% of the variance; (b) planning: the eigenvalue of this factor was 2.03 and accounted for 20.31% of the variance; (c) selective attention and inhibitory control: this factor had an eigenvalue of 1.09 and accounted for 10.9% of the variance.

We compared means of each test between groups with the nonparametric Mann–Whitney *U* test, observing that each scale has significant different scores in both groups (*p* < 0.001). Results and values are shown in detail in Table 5.

### 3.5. Convergent Validity

To examine convergent validity of the factor solution, the WAIS-IV test was used as a gold standard, and it was administered to 18% of the sample of agricultural workers (*N* = 41). From this sample, it is verified that the instruments have a significant association with the performance assessed by the gold standard test. First off, all the tests of the brief assessment tool showed a significant correlation with the full-scale IQ score. The tools that correlate with all the four factor indexes of the WAIS-IV are the adjusted FAB (FABadj) and the TMT-A. The CDT correlates only with the perceptual reasoning index, and the TMT-B correlates with the perceptual reasoning index as well, but also with the working memory index. The results of the convergent validity analysis are detailed in Table 6.

### 3.6. Test-Retest Reliability

For the estimation of the brief assessment tool's reliability in terms of its stability in time, a subsample of participants from both groups has been given the brief protocol twice, with an interval period after the first administration of about 2–4 weeks. The correlation between both periods was estimated with Spearman's rho test for each assessment tool, resulting in all the values to be significantly correlated and, hence, stable in time. Detailed values for each test and group are shown in Table 7.

### 3.7. Normative Data of the Brief Assessment Tool Stratified for Age and Educational Level

Correlations among the demographical variables (age, educational level, and household monthly income) and scores on each of the tests show that only age and educational level were correlated with the scores of all the instruments of the brief assessment tool (see Table 8). Therefore, stratification of the normative data according to these 2 variables has been performed.

In the case of the variable age, it was divided into four groups based on the age distribution of the sample, taking into account the quartiles observed. On the other hand, for the educational level, it was stratified into two groups, the first from 0 to 8 years of education, composed by workers with an educational attainment of elementary educational level, complete or incomplete. The second group is composed of participants who had between 8 and 12 years of studies, having an incomplete or complete high school educational attainment.

**Figure 3:**
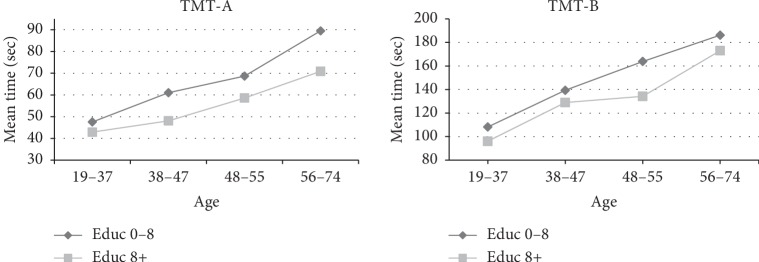
Performance in TMT-A and TMT-B, according to the four age groups and the two educational levels.

**Figure 4:**
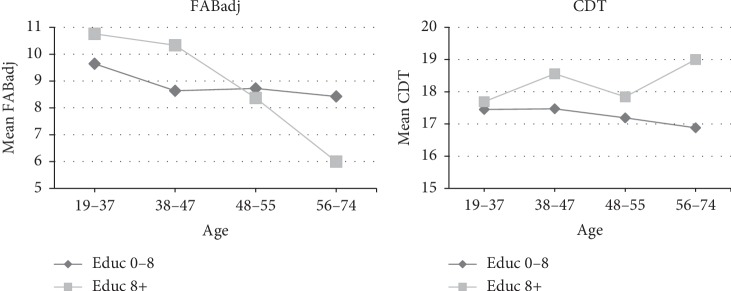
Performance in FABadj and CDT, for the four age groups and the 2 educational levels.

Figures 3 and 4 show the mean scores of the tests included in the brief assessment tool for each age group and according to the educational level, based on the stratification previously described. It is evident that in all the tests there is a detriment in the performance at older age, being more pronounced in the case of the group with lower educational level.

Table 9 shows the normative data of Chilean agricultural workers for the brief validated assessment tool, transformed into percentiles for each age group and educational level. It should be noted that for the older age group, there were no subjects with more than eight years of education. Therefore, for that group are only assumed for people with eight or less years of education.

## 4. Discussion

We have presented the process and the main results of the validation and reliability study of a brief screening scale to measure cognitive deterioration in occupational populations that are exposed to neurotoxins through the widespread use of pesticides. To date, there have been no similar studies with tools administered in Chile with these characteristics and that allow knowing in a specific way how they work with agricultural workers to monitor health and any signs of cognitive deterioration.

The main purpose has been achieved in terms of developing a tool composed of public domain tests, with no cost, simple to apply, and that could be administered by health and safety technicians due to its ease of administration and punctuation. The abridged tool complies with all the above characteristics and has also been proven to have content and construct validity with respect to the measurement of skills that are associated with those that according to the literature are among the most deteriorated in the face of chronic exposure to acetylcholinesterase inhibitory pesticides, which are the most commonly sold and used pesticides in the Chilean agricultural setting. Previous studies have shown that OP exposure impacts neurological wellbeing such as the executive functions, such as attention (sustained and focused), planning, inhibitory control, and also the visuospatial ability and processing speed [3–7]. Based on the criterion of judges, the content validity was positively verified, since the brief tests included addressed the measurement of these skills and then their pertinence was verified from the underlying factor structure with the three factors that group the different parts of the scale: (1) visuospatial skill and processing speed; (2) planning capacity; (3) selective attention and inhibitory control.

For the study, we administered the gold standard measure (WAIS-IV) to compare the scores obtained from the scale tests and evaluate their convergent validity. Our results showed that in the agricultural occupational population, the TMT-A and the FABadj are the measures most correlated with the level of global intellectual functioning (FSIQ). It is worth mentioning that all the tests included in the brief assessment tool correlate with the FSIQ or with some index of cognitive functioning measured by the Gold Standard test.

The reliability was verified both in terms of internal consistency of the tests, with values from satisfactory to high consistency, and in terms of stability over time of the measurement through test and retest. Even though all the tests of the scale showed a high and significant stability in its administration over time, the Trail Making test stands out especially in this sense, being the test with greater stability as a measure in the agricultural population.

It is expected that in the case of the CDT, the scores obtained will be from 15 points up, with a low difference expected between those cases that show deterioration compared to those who do not. In the case of the FAB, the psychometric analysis itself has led to a decrease in the number of items, because two of its original items (2 and 6) had little association with the factor solution. To administer this brief assessment tool, the items are maintained in its original composition for the CDT test and for the TMT-A and TMT-B tests. To administer the FAB, a most brief version named “FABadj” here should be used, which considers only 4 of the 6 original items (1, 3, 4, and 5). For the interpretation of the test from the norms reported here, it is advisable to follow the sequence given below:

1. Set the age group and education level of the evaluated person
2. Transform the raw score to percentile according to the normative group of the test taker
3. Report the percentiles obtained for each of the 3 factors that would be measured by each test of the scale: Visuoperceptual ability and processing speed (TMT-A, TMT-B), planning capacity (CDT), and selective attention and inhibitory control (FABadj)

A percentile of 25 or below indicates a cognitive deterioration in the specific factor measured. It should be considered that in the last normative group, the oldest, there were no subjects in the sample with more than eight years of study, so when the scale is administered to people over 56 years old, the data of the only age group available should be used to convert to percentiles. It should also be mentioned that although the TMT-B test is part of the validated scale, in our case, we observed that there were around 15% of cases where it was not possible to complete the administration because it requires knowledge of the sequence of the alphabet. It is expected that when the TMT-B is administered in an agricultural population, this type of situation could be found in workers with a very low educational level. In those cases, it would be convenient to suspend the administration of this part of the brief assessment tool in particular. Also, in cases of the TMT-B, when the test taker has a completion time of more than 300 seconds, it is advisable to suspend their administration because they have exceeded what is conventionally stated as a time limit. This would also be advisable considering the normative data obtained in the present study for agricultural occupational population since there were a minimum percentage of cases (around 1%) that had longer times of completion at that level. Therefore, it is advisable to suspend the administration of TMT-B, because in any case, its performance would be located within the 5% of worst performance according to the norm.

## 5. Conclusion

It can be concluded that the aim of this work has been met satisfactorily, establishing the validity and reliability of a brief assessment tool for the monitoring of health effects due to permanent occupational exposure to neurotoxic pesticides. Also, for the first time in Chile, there are specific norms for agricultural workers population, with instructions for administration and interpretation with ease for its potential extended use in the prevention of cognitive deterioration in the health of agricultural workers.

## Figures and Tables

**Table 1:** Neuropsychological tests and their evaluated functions.

Test	Measured functions
Clock-drawing test (CDT; Mendez et al. [24])	Visuoperceptual skills
	Self-monitoring
	Numeric sequencing
	Motor execution
	Selective attention

Frontal assessment battery (FAB; Dubois et al.[25])	Abstract reasoning
	Lexical fluidity and mental flexibility
	Motor action executive control
	Self-regulation and interference resistance
	Inhibitory control
	Environmental autonomy

Trail making test (TMT-A and TMT-B; Partington and Leiter [19])	Visuospatial skills
	Processing speed
	Attention and executive functions (cognitive flexibility)

**Table 2:** Demographic characteristics by group.

	Group	
	Agricultural (*n* = 234)	Nonagricultural (*n* = 305)
Mean age (DS)	46.26 (12.04)	41.13 (13.40)
Female percentage	20.5%	6.2%
Mean monthly income	623 USD	1044 USD

Educational level		
Illiterate	0.9%	1.0%
Primary (incomplete)	25.2%	10.8%
Primary (complete)	34.6%	16.4%
Secondary (incomplete)	12.4%	21.3%
Secondary (complete)	26.9%	44.3%
Technical/professional	0.0%	6.2%

**Table 3:** Tests scores by group, including mean, standard deviation, median, minimum, and maximum.

	Group											
	Agricultural (*n* = 234)						Nonagricultural (*n* = 305)					
	Obs.	M	DS	Med	Min	Max	Obs.	M	DS	Med	Min	Max
CDT-VS	234	9.49	1.724	10	0	11	305	9.95	1.494	10	1	11
CDT-ATT	234	3.53	0.759	4	0	4	305	3.77	0.584	4	0	4
CDT-NS	234	4.47	1.187	5	0	5	305	4.73	0.823	5	0	5
CDT total	234	17.49	3.175	19	1	20	305	18.45	2.536	19	1	20
FAB1	234	1.93	0.893	2	0	3	304	2.15	0.831	2	0	3
FAB2	234	1.96	0.978	2	0	3	304	2.19	0.853	2	0	3
FAB3	234	2.48	0.819	3	0	3	304	2.67	0.652	3	0	3
FAB4	234	2.56	0.780	3	0	3	304	2.70	0.648	3	0	3
FAB5	234	2.26	0.999	3	0	3	304	2.47	0.915	3	0	3
FAB6	234	2.95	0.282	3	0	3	304	2.97	0.256	3	0	3
FAB total	234	14.13	2.894	15	5	18	304	15.17	2.462	16	5	18
TMT-A	234	63.38	34.412	56	11	285	304	54.13	29.864	48	16	301
TMT-B	193	141.1	63.634	128	31	401	277	122.63	67.953	103	32	301

**Table 4:** Exploratory factor analysis results for the tests of the brief scale.

Items	Factor^*∗*^			Dimension
	1	2	3	
TMT-A	−0.855			Visuospatial skills and processing speed
TMT-B	−0.803			

CDT-VS		0.761		Planning
CDT-ATT		0.814		
CDT-NS		0.791		

FAB1			0.433	Selective attention and inhibitory control
FAB2	—	—	—	
FAB3			0.718	
FAB4			0.505	
FAB5			0.494	

FAB items description: FAB1: similarities; FAB2: lexical fluidity and flexibility; FAB3: sequences (programming); FAB4: conflicting instructions; FAB5: go/no go. ^*∗*^Only values of factor weight higher than 0.30 are shown.

**Table 5:** Group means comparison of performance in each test of the brief assessment tool included in the factor solution.

Test	Group		*U*	*z*	*p*
	Agricultural (exposed)	Nonagricultural (not exposed)			
CDT-VS	239.26	293.58	28492.5	−4.21	0.000
CDT-ATT	242.57	290.23	29266	−4.70	0.000
CDT-NS	256.49	279.51	32523.5	−2.60	0.000
FABadj^*∗*^	240.8	291.59	28851.5	−3.82	0.000
TMT-A	302.04	244.45	27953	−4.26	0.000
TMT-B	267.27	213.36	20598.5	−4.23	0.000

FABadj includes only items 1, 3, 4, and 5 from the original FAB.

**Table 6:** Correlations coefficients (Spearman's rho) of each test included in the brief assessment tool and WAIS' FSIQ and indexes.

	FSIQ	VCI	PRI	WMI	PSI	CDT	FABadj	TMT-A
FSIQ	1							
ICV	0.72^*∗∗*^	1						
IRP	0.76^*∗∗*^	0.28	1					
IMT	0.78^*∗∗*^	0.61^*∗∗*^	0.55^*∗*^	1				
IVP	0.63^*∗∗*^	0.34^*∗*^	0.36^*∗*^	0.29	1			
CDT	0.3^*∗*^	0.42	0.39^*∗*^	0.20	0.31	1		
FAB Adj	0.47^*∗∗*^	0.32^*∗*^	0.31^*∗*^	0.49^*∗*^	0.33^*∗*^	0.21^*∗*^	1	
TMT-A	−0.57^*∗∗*^	−0.4^*∗*^	−0.51	−0.32^*∗*^	−0.49^*∗*^	−0.29^*∗∗*^	−0.45^*∗∗*^	1
TMT-B	−0.41^*∗*^	−0.3	−0.36^*∗*^	−0.39^*∗*^	−0.2	−0.25^*∗∗*^	−0.49^*∗∗*^	−0.7^*∗∗*^

WAIS-IV indexes: FSIQ total = full-scale intelligent quotient; VCI = verbal comprehension index; PRI = perceptual reasoning index; WMI = working memory index; PSI = processing speed index. ^*∗*^=*p* < 0.05; ^*∗∗*^=*p* < 0.005.

**Table 7:** Reliability (stability) of test-retest scores in both groups.

Test and groups	Obs.	Rho	*p*
Agricultural			
CDT-total	196	0.477	0.000
CDT-VS	196	0.475	0.000
CDT-ATT	196	0.408	0.000
CDT-NS	196	0.420	0.000
FABadj	196	0.421	0.000
TMT-A	194	0.659	0.000
TMT-B	194	0.733	0.000

Nonagricultural			
CDT-total	193	0.553	0.000
CDT-VS	193	0.549	0.000
CDT-ATT	193	0.445	0.000
CDT-NS	193	0.440	0.000
FABadj	193	0.384	0.000
TMT-A	193	0.726	0.000
TMT-B	177	0.660	0.000

**Table 8:** Correlations among age, education, and household income with the tests.

	Age	Education	Family income	CDT	FABadj	TMT-A
Age	1					
Education	−0.60^*∗∗*^	1				
Household income	−0.06	0.06	1			
CDT	−0.16^*∗*^	0.22^*∗∗*^	0.04	1		
FABadj	−0.35^*∗∗*^	0.37^*∗∗*^	0.06	0.12	1	
TMT-A	0.51^*∗∗*^	−0.40^*∗∗*^	−0.08	−0.24^*∗∗*^	−0.39^*∗∗*^	1
TMT-B	0.45^*∗∗*^	−0.39^*∗∗*^	−0.08	−0.21^*∗∗*^	0.46^*∗∗*^	−0.65^*∗∗*^

^*∗*^ *p* < 0.05; ^*∗∗*^*p* < 0.005.

**Table 9:** Percentiles for the brief assessment tool for each normative group.

Percentile	Education 0–8 years				Education 8 + years			
	CDT	FABadj	TMT-A	TMT-B	CDT	FABadj	TMT-A	TMT-B
19–37 years old (*n* = 56)								
95	20	12	33	72	20	12	22	53
90	20	12	33	72	20	12	26	62
75	19	12	34	75	20	12	32	73
50	19	10	39	94	19	11	50	102
25	17	8	52	124	16	10	58	128
10	10	6	67	143	12	9	63	177
5	9	6	67	143	9	8	69	195

38–47 years old (*n* = 63)								
95	20	12	30	81	20	12	27	54
90	20	12	33	90	20	12	30	59
75	19	11	43	109	20	12	37	81
50	18	9	55	135	19	11	45	121
25	18	7	69	183	18	9	57	146
10	12	3	87	248	14	7	80	216
5	6	2	107	294	13	5	87	318

48–55 years old (*n* = 62)								
95	20	11	39	65	20	11	33	73
90	20	11	42	93	20	11	37	79
75	19	11	49	114	20	11	48	107
50	18	9	62	149	19	8	58	129
25	16	8	79	199	17	6	64	168
10	13	5	93	251	14	5	85	194
5	10	2	109	274	12	4	85	194

56–74 years old (*n* = 53)								
95	20	12	36	95	–	–	–	–
90	20	11	45	104	–	–	–	–
75	19	10	58	116	–	–	–	–
50	18	9	79	168	–	–	–	–
25	16	7	104	220	–	–	–	–
10	12	3	143	311	–	–	–	–
5	7	3	197	370	–	–	–	–

## Data Availability

The datasets are available from the first corresponding author on reasonable request. The ethical approval granted to the authors does not allow the publication of the dataset online. If readers would like to reanalyse the dataset, additional ethical approvals (on an individual user and purpose basis) will be required.
